# E-Portfolio as an Evaluative Tool for Emergency Virtual Education: Analysis of the Case of the University Andres Bello (Chile) During the COVID-19 Pandemic

**DOI:** 10.3389/fpsyg.2022.892278

**Published:** 2022-06-17

**Authors:** Rubén Rodriguez, Lorena Martinez-Ulloa, Carolina Flores-Bustos

**Affiliations:** School of Speech Therapy, Faculty of Rehabilitation Sciences, Universidad Andrés Bello, Santiago, Chile

**Keywords:** E-portfolio, formative evaluation, higher education, students perception, pandemic (COVID-19)

## Abstract

The pandemic had serious implications for university education, specifically due to the transition from face-to-face teaching to online methodologies. This article analyzes the perception of students undergoing speech therapy from a Chilean University about the E-portfolio incorporation as an evaluative tool during the emergency virtual teaching due to the COVID-19 pandemic. From quantitative research, a survey of 38 questions based on Likert scales was applied to 108 penultimate year undergraduate students. The survey demonstrated that there is an improvement in the methodology and teaching support, as well as in the creativity and professionalism of the students.

## Introduction

Before the arrival of the COVID-19 pandemic, higher education had to shift abruptly from a face-to-face workspace to a virtual workspace (Revilla-Cuesta et al., 2021; Telyani et al., 2021; Tang et al., 2021). And Chile was no exception, as the pandemic had affected the different educational levels, particularly the higher education sector. For instance, the authors Rivera Olguin et al. (2021) along with a group of university teachers evidenced that due to the COVID-19 pandemic, most of the teachers revealed a lack of knowledge regarding the management of digital technologies. In addition, due to the non-existence of an objective model of virtual education, it brought about blind spots in content, methodology, and evaluation. Other studies have shown difficulties related to the interaction methods with the students, laboratories, and professional practices (Revilla-Cuesta et al., 2021). In fact, these authors agree that despite the problems faced in teaching practices, some continue with the same traditional performances, but behind a computer screen. During the pandemic, the teachers don't have time to prepare themselves for the teaching transition, which involves shifting from traditional teaching to an online teaching platform and transforming their practices based on the learning, motivations, interests, and academic commitments of students (Toquero, 2020; Daumiller et al., 2021). So, the present study aims to contribute to the development of the field of study regarding the perceptions that university students have about their educational experience which due to the pandemic had to migrate to online learning environments. Although research have been carried out on the use of portfolios and their impact on the training outcomes of university students (Welsh, 2012; Riquelme et al., 2011; Revilla-Cuesta et al., 2021), only a few studies in Chile formatively evaluated the perceptions of students in the implementation of an E-portfolio model in the online context. Thus, this study justifies the desire to search for the perceptions presented by students at the university level about the educational experience encountered by them during the implementation of the E-portfolio as a model for a formative evaluation. This was done through the introduction of the course “Design of Management Project in Speech Therapy,” which due to the COVID-19 pandemic was made to shift from a face-to-face learning environment to an online mode. In addition, the following questions must be solved: What are the benefits of E-portfolio to students' learning? Is E-portfolio an appropriate tool for the formative assessment of an online course? Thus, by answering these questions, this study proposes a formative evaluation model, through E-portfolio, and its implementation in an online context to support learning in higher education.

## Theoretical Framework

### Effects of COVID-19 on Learning

Although the concept of online teaching is not new, the COVID-19 pandemic has strongly promoted this teaching methodology around the world. Due to the pandemic, students of all educational levels could no longer attend school, challenging institutions to virtualize their academic activities while maintaining their quality (Toquero, 2020). These emergencies, along with associated difficulties and obstacles, impacted teachers in terms of content organization, teaching methodology, and online assessment. Simultaneously, it had a psychological impact on students and on the performance and objectives linked to their learning, which means that it caused a significant impact on their cognitive and emotional processes (Schiff et al., 2020; Rahiem, 2021).

Moreover, while some studies have shown that the transition from face-to-face teaching to online teaching is a good practice, others have shown the opposite results. For example, researchers Daumiller et al. (2021) in a study on attitudes, exhaustion, and cognitive commitment among teachers and students showed that the teaching and learning approaches in the online transition were positive. In addition, other studies showed that in times of pandemic situations, communication has been ineffective in student–teacher and teacher–student interaction, which translates into challenges for effective learning (Telyani et al., 2021).

The above-mentioned difficulties, combined with the restrictions of not being able to relate to friends and family, affect the motivation and interaction of students, -leading them to failure in their learning in the long term. According to Bandura (1989), the process of socialization between student and teacher must be effective and affective, such that it is possible to mobilize quality learning that positively impacts the cognitive processes. At the same time, it is emphasized that the technology used in an online environment does not determine meaningful learning (Ahmed et al., 2020).

### E-Portfolio and Evaluation

An E-portfolio refers to a collection of work that each student develops through collecting, selecting, organizing, reflecting, and presenting their understandings and growth over time. E-portfolios have been increasingly used in higher education as a facilitator of formative learning and assessment (Rodríguez et al., 2013). In addition, an important component of the E-portfolio is the student's reflection on the work of the individual activities, as well as the general reflection on the set of activities contained in the portfolio (Paulson and Paulson, 1991; Nahadi and Siswaningsih, 2021). Other authors underline that a digital portfolio is a powerful tool that includes active methodologies that focus on promoting permanent and autonomous learning in students, and it is a platform where they can deploy transversal skills and critical thinking (Sarwandi and Wibawa, 2022).

An exploratory study on the perceptions of 40 university students regarding the integration of E-portfolios showed that they valued this experience in terms of promoting learning communities (Nahadi and Siswaningsih, 2021). Another study on the use of these learning tools showed that they facilitate student collaboration, peer feedback, stimulation of reflection, professional development, and formative assessment (Bolliger and Shepherd, 2010). Along the same lines, the authors Nahadi et al. (2015) agree that the evaluation of the E-portfolio supports the learning process of students and that it evaluates the data compiled from the activities developed during the training process, both cognitive and affective. In fact, feedback in formative assessment tends to improve students' learning outcomes, as well as their mental processes about the content delivered to them by the teacher.

It should be noted that the use of an E-portfolio as an evaluation model in the university educational field is a topic that has not been well researched. However, various investigations have highlighted its potential to visibly analyze the student's performance in an integral way, which is not possible to measure in a concrete way through tests (Barret, 2010; Firman et al., 2018; Nahadi and Siswaningsih, 2021). In summary, the E-portfolio is a didactic strategy and a complement to the professional and academic development of students from a methodological and evaluative perspective, allowing them to develop reflective and creative practice, collaborating working skills identity and the use of technology Apps.

## Method and Context

### Context of University Andrés Bello

This study is carried out at the School of Speech Therapy, Andrés Bello University of Chile. The study program includes a set of subjects that are linked to the management of entrepreneurship and innovation projects, among which is the subject “*Management Project Design in Speech Therapy*,” which we use to intervene with the proposal of the use of E-portfolio. This subject pays tribute to the scope of the graduate profile “*Research projects and management in speech therapy*,” specifically in the learning outcome: “To Develop a speech therapy management project with its respective ethical considerations, which contributes to the development of the discipline for the benefit of people and their community.”

Specifically, the purpose of the subject is to integrate skills and knowledge built throughout their training, which allow the student to develop knowledge strategies for the design of management projects in speech therapy, keeping in view the community problems and the concordant theoretical ethical framework. At the end of the course, the student is going to be able to determine and identify problems in the areas of speech therapy related to management, and design management projects that are relevant and essential for the community and its environment (Universidad Andrés Bello, 2019).

### Methodological Design

The objective of this study is to analyze the perception that university students have about their educational experience at the time of the implementation of a formative evaluation model. This was done based on the implementation of the E-portfolio during “Project Design of Management in Speech Therapy” course, which was virtualized full-time due to COVID-19.

First, the study subjects were organized into six sections of approximately 20 students each, and for the implementation of the E-portfolio for 4.5 months, each section in charge of a teacher was organized into groups of five students. They were asked to develop a project that would be organized and presented through the portfolio on a weekly basis to the teacher, who, on the one hand, would give feedback in writing through a workshop and, on the other hand, would provide summative assessment in the eighth and fifteenth week. In addition, self-assessment and co-assessment rubrics were organized and designed for the evaluation.

The research adopts a quantitative approach, and includes a descriptive design with cross-sectional temporality. A Likert scale survey was applied to the entire population of the study at the end of the course. In addition, and as a complement, it was decided to apply the following open questions taken from the author Welsh (2012): What are the best aspects of the evaluation and feedback during the use of the E-portfolio? What are the best aspects of evaluation used in the E-portfolio methodology? and What aspects of adaptation in the students' learning did the teacher consider during the application of the E-portfolio?

### Participants

In total, 127 students enrolled in the subject of Management Project Design in Speech Therapy at the Andrés Bello University. This study was carried out during the first half of 2021 in an online mode. All students who completed the subject and did not exclude the course before the end of the semester were included in the study population. Finally, 108 participants answered the survey voluntarily, corresponding to a confidence level of 95% and a margin of error of ±3 in relation to the representativeness of the sample on the universe.

### Data Collection Procedures and Techniques

To measure students' perceptions of the evaluative experience using E-portfolios, a survey of the perception of the methodological use of the portfolio designed and validated by Riquelme et al. (2011) was used. It should be noted that this was adapted and contextualized to the nature of the subject, based on a methodology of theory created through the focus group technique by students, teachers, and specialists in the area. The level of agreement or disagreement among the respondents was measured by means of a Likert Scale graduated from 0 to 4, where 0 corresponds to completely disagree, 1 disagree, 2 you are unsure or have no opinion, 3 agree, and 4 completely agree; in the case of negative statements, the graduation is the other way around.

The reason for selecting this instrument is because it addresses all the study variables, such as students' learning, organization and evaluation, methodology and teaching support, creativity, and integration. Furthermore, of a total of 43 statements raised in the original survey, five were eliminated for reasons of context and disciplinary nature, leaving only 38 statements distributed in each of the variables mentioned. In addition, as part of the objectives, the reliability of the survey was measured by the Cronbach method, and the internal consistency of the data was evaluated through the calculation of Cronbach's alpha coefficient, using the SPSS statistical software (George and Mallery, 2003).

Table 1 shows that, in general, the instrument has an acceptable internal consistency with a mean Cronbach alpha > 0.7. However, the variable organization and evaluation presents an alpha < 0.6, which means that it is questionable in terms of the number and types of statements that are responsible for measuring this construct.

**Table 1:** Cronbach's alpha by study variable.

**Study variable**	**Cronbach alpha**	**Distribution of statements by variable N**°****
Students' learning	0.976	23
Organization and evaluation	0.597	4
Methodology and teaching support	0.889	7
Creativity and integration	0.869	4

The survey was applied virtually to the representative sample of students, who were distributed in six different sections with a maximum of 20 per group; each section was headed by a teacher.

### Analysis Method

To obtain the information, a Likert scale survey was used. Subsequently, the data obtained were analyzed in the SPSS22 quantitative analysis software. To obtain the information regarding the use of the E-portfolio, three questions were administered to all the students. Then, the audio format of their statements was transcribed to obtain relevant information units on our study variables through the qualitative analysis software VERBI Software (2007).

Next, we categorized the information units, which meant assigning them codes to identify the subject and category of study, and the latter information was obtained from the variables considered in the quantitative instrument. In Table 2, the codes and categories used are presented.

**Table 2:** Codes of the study categories.

**Categories**	**Definition**	**Codes**
Students' learning	Development of the learning through use of E-portfolio and its teaching accompaniment.	SL
Organization and evaluation	Organization and purpose of the evaluation.	OE
Methodology and teacher support	The use of active methodologies in the implementation of E-portfolio as a model to activate a formative evaluation through a teaching accompaniment.	MTS
Creativity and integration	Organization and integration of the information through the use of E-portfolio.	CI

For example (Table 3) *S*25_13_*OE* the subscript indicates that proposition 1 comes from information unit 3 of student 25 corresponding to the Organization and Evaluation (OE) study category.

**Table 3:** Elaboration of propositional units.

**Information units**	**Propositional units**
*S*25_3_*OE* the very kind teacher helped us a lot in our project, even when we needed a meeting, he helped us very quickly and with great disposition.	*S*25_13_*OE* the very kind teacher helped with our project. *S*25_23_*OE* the teacher with great willingness helped us through meetings.

These propositional units were exposed in the main results associated with the study variables: students' learning, organization and evaluation, methodology and teacher support, and creativity and integration.

## Results

The details of the information obtained from the survey applied to 108 students are given in the following text. The results are presented according to the study variables: students' learning, organization and evaluation, methodology and teaching support, and creativity and integration. On the vertical axis of the graph, the value 0 corresponds to completely disagree; 1, disagree; 2, you are unsure or have no opinion; 3, agree; and 4, completely agree, while the horizontal axis shows the statements related to each of the variables considered in this study.

### Students' Learning

**Figure 1:**
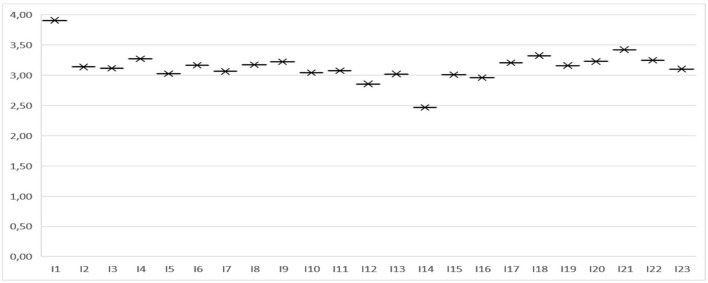
Mean of the student's learning variable.

Figure 1 clearly shows that the students show a highly marked tendency toward maintaining the E-Portfolio for future courses, as they very much agree that it was well organized and, therefore, is very useful for the development of skills associated with the responsibility of solving a problem located and contextualized in the society. On this point, participants agree that the implementation of this tool is something they enjoyed because it gives them meaning in their professional and internship development.

Likewise, the participants completely agree that the E-portfolio had a positive impact on the development of their learning, specifically by a good accompaniment from the teacher, and that their feedback was adequate and effective throughout the process of preparing the management project. This finding highlights that students perceive that the E-portfolio, besides promoting autonomous learning, is a good methodology to promote a fair formative evaluation. In short, the average mean value of the students' learning variable is 3.14, which on the Likert scale corresponds to an acceptable internal consistency.

Based on the above-mentioned results, most students declare that the E-portfolio, as a methodology, is effective in successfully carrying out the development of a project (Table 4), which is facilitated through permanent and timely feedback from the teacher. On this last point, they declare the following points:

*S*1_12_*SL* *The feedback from the teacher allowed our project to improve…*

*S*43_11_*SL* *The observations and advice given by the teacher generate a constructive approach…*

*S*12_13_*SL* *It is useful to learn how to carry out management projects and to organize yourself as a group…*

**Table 4:** Statements in students' learning.

**Code**	**Statements**
*S*45_12_*SL*	*The E-Portfolio is very useful to create a successful project…*
*S*4_23_*SL*	*It is useful to learn how to carry out management projects and to organize yourself as a group…*
*S*18_22_*SL*	*It's an opportunity to change so you don't make the same mistakes again…*
*S*29_21_*SL*	*The feedback from the teacher allowed our project to improve…*
*S*105_21_*SL*	*The E-Portfolio helped us a lot to be able to complete our project…*
*S*13_21_*SL*	*The creation of the project through E-Portfolio was very favorable for learning…*
*S*100_21_*SL*	*I think the formal evaluation is unnecessary…*
*S*9_21_*SL*	*It is favorable to discuss different points of view in the group…*
*S*107_11_*SL*	*I really enjoyed working in a group and my colleagues gave their opinion about our work*.

In addition, most of the students declare that during the implementation of the E-portfolio, the activities were carried out in an objective time, so that they were completed without many difficulties, emphasizing that everything was carried out step by step through a reflection mediated by the teachers. They issue the following statements:

*S*2_13_*SL* *The work done and the tools delivered will be very useful for future projects*.

*S*17_13_*SL* *Honestly, I thought it was a very good and motivating idea to develop the branch throughout the semester*.

Finally, they declare that they value teamwork, particularly for implementing a different method of evaluation, which was different from the traditional ones.

### Organization and Evaluation

**Figure 2:**
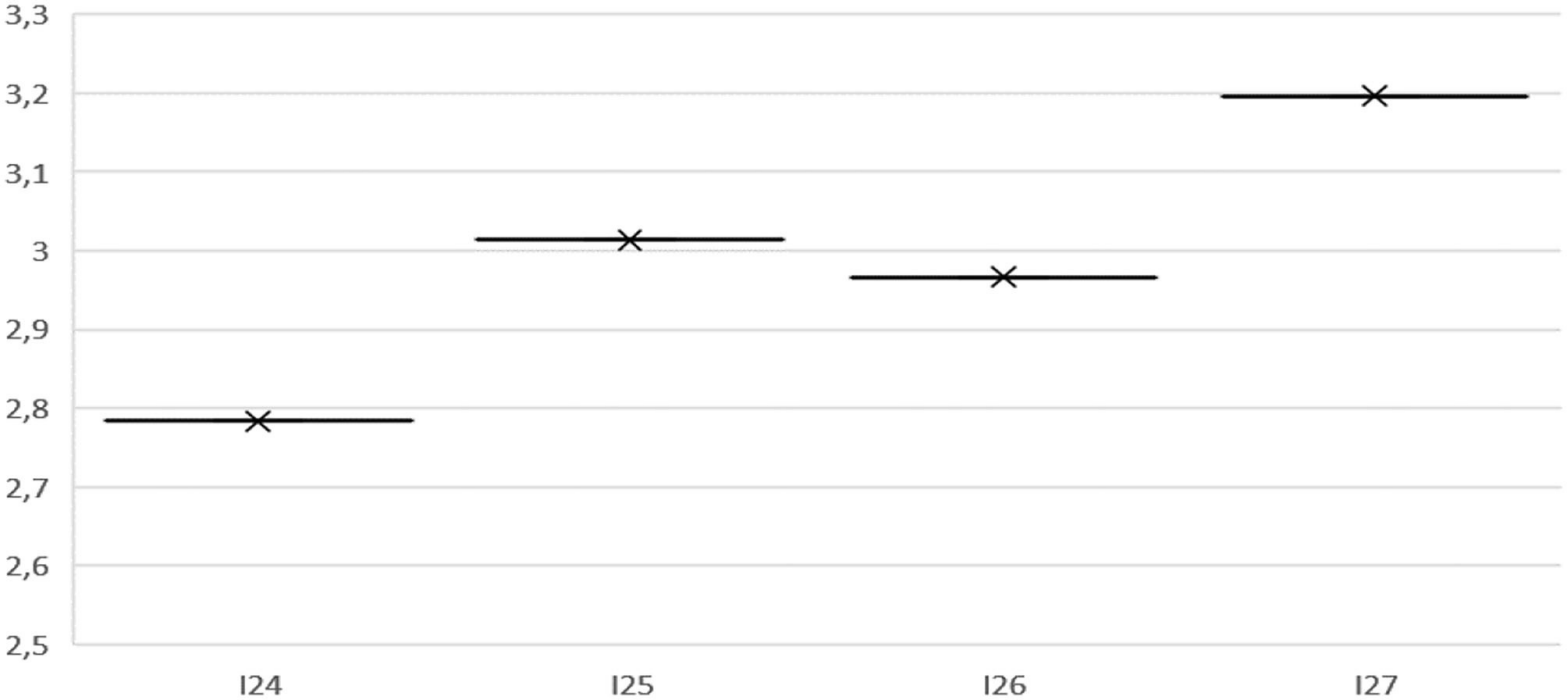
Mean of the variable organization and evaluation.

It is shown in Figure 2 that the mean of the organization and evaluation variable is 2.9 on the Likert scale, which corresponds to agree, that is, the participants agree that the instructional part of the E-portfolio is clear. However, on the one hand, they perceive that peer-to-peer copying of E-portfolio becomes a problem, while on the other hand, they agree that they had protected time for its development.

The results obtained for the variable organization and evaluation, through the survey applied to students, is consistent with the statements made by them (Table 5). For example, the majority declares that although there is no individual evaluation, since all the members do not contribute to the collective work, the co-evaluation offered them the opportunity to reflect and integrate the opinions of their peers who were responsible for the development of the projects. Students issue the following statements:

*S*98_32_*OE* *All aspects of evaluation were very consistent…*

*S*10_23_*OE* *The self-assessment and co-assessment I feel were very efficient…*

**Table 5:** Statements in organization and evaluation.

**Codes**	**Statements**
*S*103_11_*OE*	*Self-evaluation is very beneficial and process evaluation can show the progress that occurs during the process*.
*S*19_22_*OE*	*The student sees the irresponsibility of certain classmates…*
*S*36_13_*OE*	*Entire groups disconnect and disappear from the class when progress has to be shown and the teacher does not say anything or does not evaluate in a fairer way*.
*S*15_21_*OE*	*Everyone in the group got the same grade, which is unfair to the colleagues who worked the hardest*.
*S*65_31_*OE*	*The teacher was always responsible with the evaluation times and with the feedback of the works*.
*S*34_12_*OE*	*The teacher adjusted to our times to be able to make corrections*.
*S*91_32_*OE*	*Due to the form of evaluation, it became easier to understand the mistakes*.

In addition, students state that teachers were very responsible with the delivery times of feedback and evaluations, giving them enough time to carry out improvements in their projects. In fact, they emphasize that in special cases, the teacher adjusted the time according to the needs and/or characteristics of the students. In this, they declare that:

*S*48_12_*OE* *The very gentle teacher helped us a lot in our project and its evaluation…*

*S*108_11_*OE* *We needed a meeting and he helped us very quickly and with a lot of disposition…*

*S*21_22_*OE* *The best thing about the evaluation and feedback is that the teacher knew what our work was like as a team…*

### Methodology and Teacher Support

**Figure 3:**
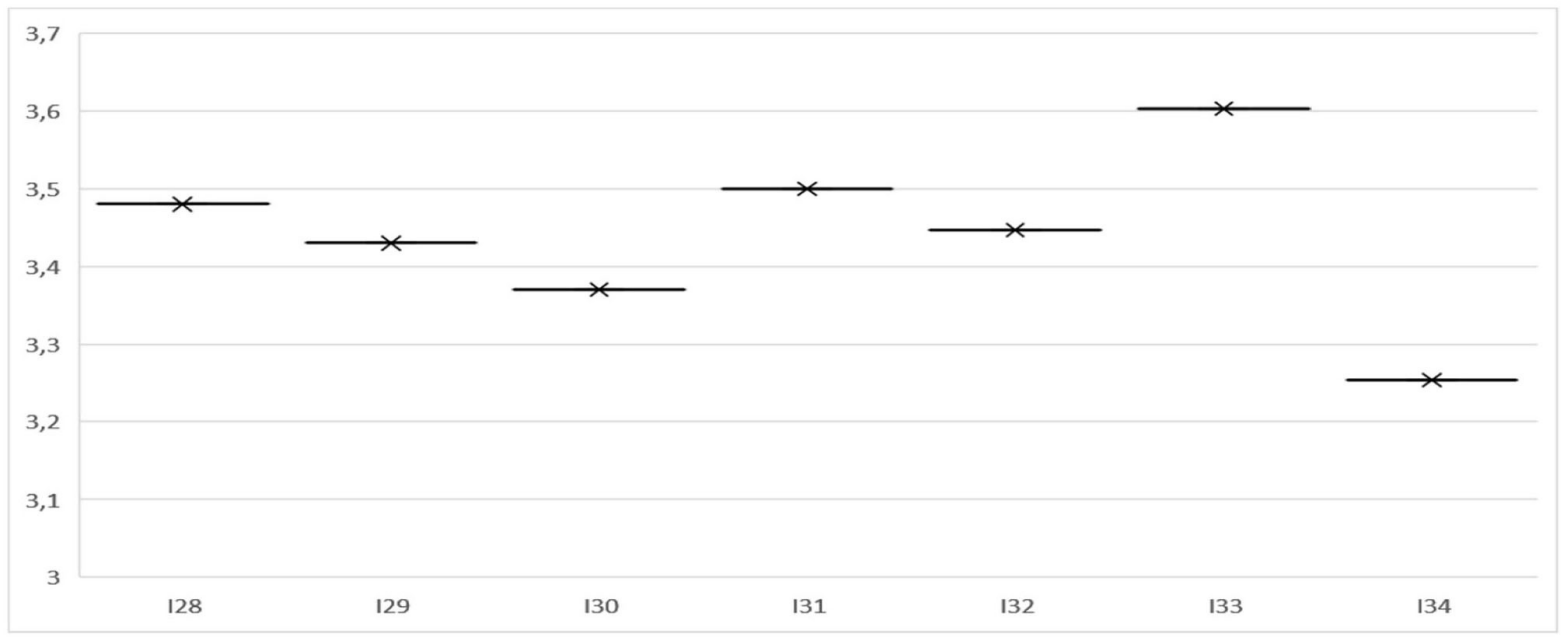
Mean of the variable methodology and teaching support.

From Figure 3, the perception that students have about their teachers is that they agree that the analysis of the management project is focused on the diagnosis of the community. In line with this, they agree that they act according to their individual characteristics, which motivates them and, therefore, actively participate in the development of their learning with responsibility and progressive autonomy. In addition, they agree that material and technological support is adequate, something that facilitated coordination between the activities of the E-portfolio and the remaining activities of the semester. Finally, it should be noted that the participants in this study agree that they had opportunities to improve those aspects evaluated as deficient in their feedback, and they agree that E-portfolio learning is active. In short, the mean of the variable methodology and teaching support is 3.47 on average, which on the Likert scale corresponds to an acceptable internal consistency.

In addition, the statements made by the students regarding methodology and teaching support (Table 6) coincide with what was evidenced in the surveys. For example, most state that teachers were constantly motivating them during the process of their learning, so they had many opportunities to improve all those aspects evaluated as deficient after feedback. On this, they state that:

*S*101_21_*MTS* *The teacher took the time to review together with the group every detail of the project…*

*S*20_11_*MTS* *The great adaptability of the teacher to situations that emerged…*

*S*50_13_*MTS* *The fact that it was schedules for each group when presenting the doubts was undoubtedly an aspect that motivated me to carry out the work efficiently…*

**Table 6:** Statements in methodology and teaching support.

**Codes**	**Statements**
*S*70_22_*MTS*	*He always carried out an evaluation in a cordial way and resolved doubts…*
*S*54_11_*MTS*	*Always any questions when sending an email or talking to him was immediately…*
*S*56_22_*MTS*	*The teachers are committed to the students' projects…*
*S*24_11_*MTS*	*The feedback from the teacher is quite complete and helps to improve the project…*
*S*92_11_*MTS*	*Thanks to this feedback you can get a more generalized view of what our performance is like…*
*S*75_12_*MTS*	*The feedback from our project helped us a lot to improve all the points of our project…*
*S*39_13_*MTS*	*Each activity was carried out class by class…*
*S*7_21_*MTS*	*The empathy of the teachers regarding the technical unforeseen events that we presented as a group was good…*

### Creativity and Integration

**Figure 4:**
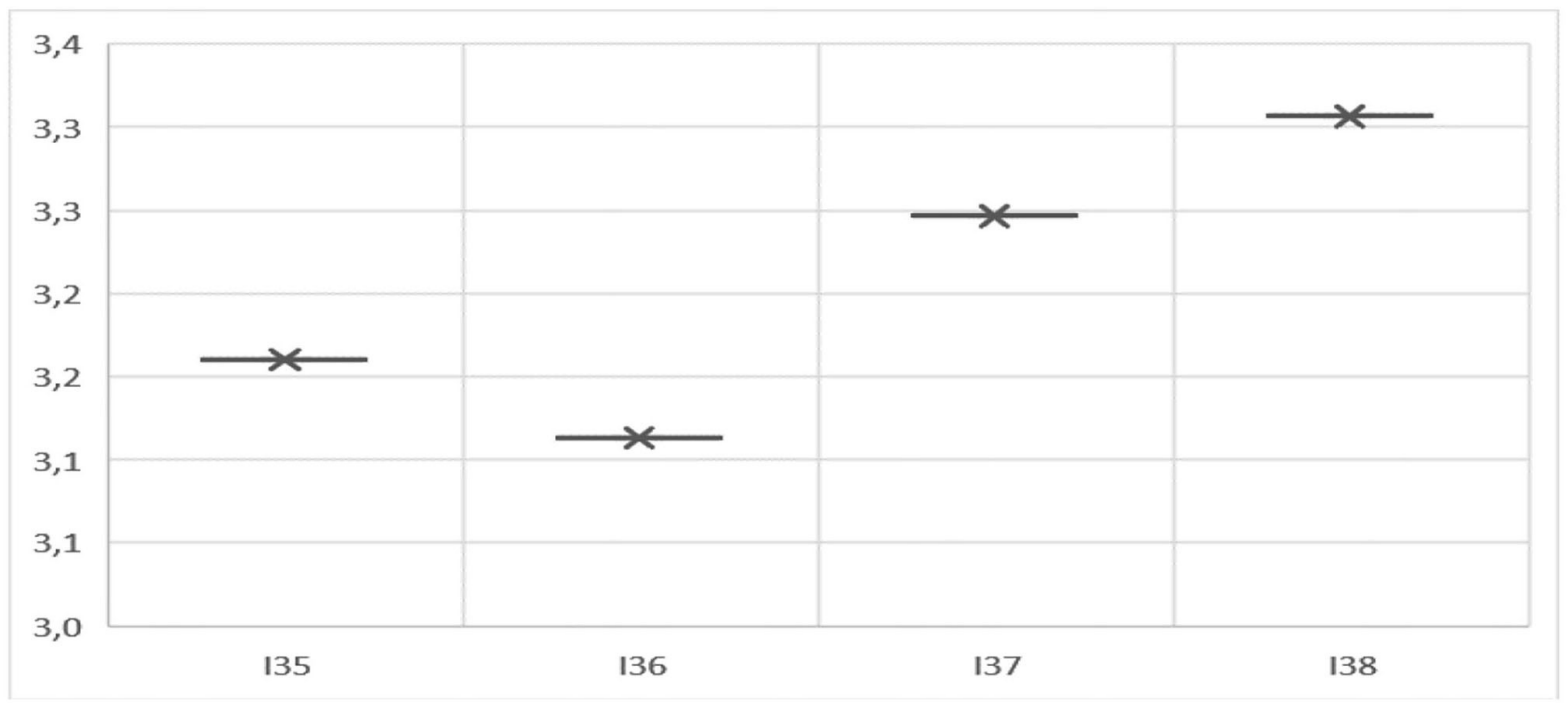
Average of the variable creativity and integration.

The participants surveyed agree that working with E-portfolio does not translate into the mere transcription of the subject but allows them to develop creativity in terms of the different ways of ordering or configuring information in terms of their cognitive processes. They also agree that the implementation of this, as a methodology for the elaboration of a project, allowed them to mobilize and apply previous and new knowledge to real community problems based on epidemiological variables and social, territorial, and ethical aspects. In short, the mean of the creativity and integration variable is 3.21 on average, which on the Likert scale corresponds to an acceptable internal consistency (Figure 4).

The main results obtained from the respondents in creativity and integration are consistent with the statements made by most participants (Table 7). In this regard, most of them declare that the E-portfolio methodology implemented is far from traditional teaching, since it forces them to review, reflect, and correct each of the activities associated with the projects. In addition, a majority agree that the methodology allowed them to successfully deploy their previous knowledge, thus obtaining effective learning.

**Table 7:** Statements on creativity and integration.

**Codes**	**Statements**
*S*49_11_*CI*	*… a satisfactory method by having the “obligation” to review and correct the previous works obtaining a good result…*
*S*53_21_*CI*	*I learned a lot about how and what a project should implement considering aspects that I did not have before…*
*S*66_12_*CI*	*The contents learned and the different topics that were imparted should have a solution through the projects…*
*S*78_13_*CI*	*The E-Portfolio helped to learn and develop self-criticism skills…*

## Discussion and Conclusion

### Students' Learning

Our results show that in the students' learning variable, they present a favorable perception regarding the use of the E-portfolio as an evaluation strategy, results similar to those published by a previous study (Welsh, 2012; Nahadi et al., 2015; Makransky et al., 2019). In this context, through the use of the E-portfolio, students manage to present their way of thinking, enriching it through the exchange of ideas among their peers and professors in charge of the groups (Paulson and Paulson, 1991; Rodríguez and Aguado, 2009). This is consistent with the findings of a previous study (Welsh, 2012), where students value feedback both from their peers and teachers (Revilla-Cuesta et al., 2021). In addition, the decision to offer feedback to small groups of students during the implementation of the E-portfolio caused it to be distributed more evenly throughout the virtual workshops and enabled synchronous meetings with teachers. The E-portfolio had a positive impact on the development of students' learning, because they enjoyed its implementation and believed that it gave them meaning in their professional and internship development. We consider in this context that the use of E-portfolios implies a work methodology on the part of the teacher that allows them to specify the results and learning outcomes, showing a broad pedagogical sense. In addition, we consider introducing E-portfolios in the processes of teaching and learning involved in each of the professional careers, and it is the primary task of universities/institutions, where teachers and trainers are the main promoters.

### Organization and Evaluation

The students in this study perceive that the time used for the development of the E-portfolio is adequate. However, students are concerned that the tasks will be copied by their peers. In this sense, we agree with Sarwandi and Wibawa (2022) who state that the use of E-portfolios as a model to promote formative assessment has good scope and potential in integrating knowledge, skills, and attitudes (Makransky et al., 2019). In this regard, in this study, the students have a very positive perception regarding the group evaluation carried out by the teachers in an effective and timely manner (Welsh, 2012). However, it is evident in this study that there was no context of individual evaluation that would allow showing those students who were contributing ideas collectively; this is under the assumption that formative evaluation with permanent and significant feedback increases motivation and participation of students to continue learning. In this context, Nahadi et al. (2015), in a study of the implementation of an E-portfolio for the development of critical thinking skills in university students, showed that the number of tasks is excessive, and the institutions are not used to evaluating them. The variable organization and evaluation received the lowest average scores in the study, noting that, although there is a favorable perception regarding the organization of the model proposed by this experience, it is relevant to reinforce aspects of individual evaluation and monitoring of student's participation in group work.

In brief, we believe that beyond the organization of the evidence of learning through the use of the E-portfolio, the interaction between the student and teacher must be taken into account, so that the evaluation acquires great relevance in the permanent improvement of the learning acquired by the students. To achieve this, a teacher and a student are required to be willing to get involved in the learning process and in the use of the E-portfolio in a teaching and learning situation accompanied by active methodologies and various evaluation instruments (Rodríguez et al., 2013).

### Methodology and Teacher Support

The study variable methodology and teaching support received the highest average scores, where the high assessment of the students about the proposed model stands out in the context of the COVID-19 pandemic, where the exhaustion and commitment of the students were strongly supported by teachers (Telyani et al., 2021). On this point, the authors Daumiller et al. (2021) point out that the transition from face-to-face teaching to online teaching necessarily requires teachers to design high-quality teaching material, should know how to virtually communicate content to their students, and how to improve teacher–student and student–teacher interaction (Revilla-Cuesta et al., 2021). Hence, students reflect on the development of their learning in a consistent and adequate manner, such that it gives them a conceptual, procedural, and attitudinal basis to apply in various contexts.

In this study, the perception that the students have about the work developed by the teachers was very positive, showing that they act considering the individual characteristics of their students, which motivates them and develops autonomy in their learning. This coincides with some authors, who point out that before implementing the E-portfolio, the context of the students must be considered (Revilla-Cuesta et al., 2021).

Finally, the perception that students have about the activities of the E-portfolio is that they were constantly presented with opportunities to reflect on those aspects of learning that were wrongly understood, and as we have already mentioned in other variables of the study, this is due to the implementation of a formative evaluation (Nahadi et al., 2015).

### Creativity and Integration

The results of this study show that the respondents present an acceptable perception of the use of the E-portfolio in the context of the COVID-19 pandemic, since it allowed them to develop cognitive creativity in terms of the different ways of configuring the information in the E-portfolio and discussing it both with their peers and with the teacher. And in this regard, like Martin Daumiller, we agree that the correct motivations and attitudes in this pandemic would allow students to recognize the challenges in their teaching process as opportunities for improvement.

In brief, the implementation of the E-portfolio for the students of this study meant significant participation in a virtual platform and included the following positive outcomes: a greater awareness of their own learning, an increased sense of responsibility for their own learning (being the protagonist of the activities that are developed), collaborative construction of knowledge by sharing materials and reflections, a shared and fairer evaluation including external evaluation of the teacher, co-evaluation among peers and self-evaluation, and greater awareness and knowledge about the learning process and its development over time with permanent monitoring and feedback.

We can conclude that despite the COVID-19 pandemic, the E-portfolio model proposed to develop active methodologies and formative evaluation has a positive impact on students' learning.

So, this study suggests that the educational institutions should develop programs to prepare students not only for the workforce but also to face unexpected situations, such as the COVID-19 pandemic, and take advantage of these interventions to highlight teaching practices and improve them considering social, political, technological, and/or cultural changes.

In summary, and as a result of the COVID-19 pandemic, the curricula were unexpectedly more flexible, so this intervention in the subject “Management Project Design in Speech Therapy” suggests that institutions appeal to rethink the disciplines included in a curriculum in terms of the amount of content and its possibilities of being virtualized.

For future research, it is necessary to review the measurement instrument for the incorporation of other questions that delve into aspects of transversal skills and their appropriation through the proposed pedagogical strategies, such as teamwork, information resource management, and ability to self-learning. Likewise, we are aware that within the measures used there are inherent limitations of the quantitative studies, therefore, for future investigations we suggest, for one side, the incorporation of variables that have direct implication in the development of transversal skills and in the other side, associated variables in formative effective evaluation (Nahdi et al., 2021).

## Data Availability Statement

The raw data supporting the conclusions of this article will be made available by the authors, without undue reservation.

## Ethics Statement

The studies involving human participants were reviewed and approved by the Universidad Andrés Bello. The participants provided their written informed consent to participate in this study.

## Author Contributions

RR, LM-U, and CF-B contributed as supervisor of the study, revised, read, approved the draft, and the submitted version of the manuscript. All authors contributed to the article and approved the submitted version.

## Funding

Research reported in this publication was supported by the Universidad Andrés Bello.

## Conflict of Interest

The authors declare that the research was conducted in the absence of any commercial or financial relationships that could be construed as a potential conflict of interest.

## Publisher's Note

All claims expressed in this article are solely those of the authors and do not necessarily represent those of their affiliated organizations, or those of the publisher, the editors and the reviewers. Any product that may be evaluated in this article, or claim that may be made by its manufacturer, is not guaranteed or endorsed by the publisher.
